# HDGF在手术切除非小细胞肺癌中的异常表达及其在预测预后中的意义

**DOI:** 10.3779/j.issn.1009-3419.2011.03.06

**Published:** 2011-03-20

**Authors:** 军 张, 娟 祁, 艳 郭, 义 郭, 伟能 富, 宝森 周, 广平 吴, 立波 韩, 安光 何

**Affiliations:** 1 110001 沈阳，中国医科大学附属第一医院，中国医科大学肺癌中心 China Medical University Lung Cancer Center, the First Hospital of China Medical University, Shenyang 110001, China; 2 110001 沈阳，中国医科大学附属第一医院，肿瘤分子靶向治疗研究室 Department of Molecular Targeted Therapeutics, Cancer Institute, the First Hospital of China Medical University, Shenyang 110001, China; 3 110001 沈阳，中国医科大学附属第一医院，胸外科 Department of Thoracic Surgery, the First Hospital of China Medical University, Shenyang 110001, China; 4 110001 沈阳，中国医科大学附属第一医院，病理科 Department of Molecular Genetics, China Medical University, Shenyang 110001, China; 5 110001 沈阳，中国医科大学分子遗传学教研室 Department of Epidemics, China Medical University, Shenyang 110001, China; 6 110001 沈阳，中国医科大学流行病学教研室 Department of Pathology, the First Hospital of China Medical University, Shenyang 110001, China

**Keywords:** 肺肿瘤, 肝癌衍生生长因子, 生存分析, 预后, Lung neoplasms, Hepatoma-derived growth factor, Survival analysis, Prognosis

## Abstract

**背景与目的:**

在前期研究中发现肝癌衍生生长因子（hepatoma-derived growth factor, HDGF）在A549、H226等非小细胞肺癌（non-small cell lung cancer, NSCLC）细胞中明显高表达，在促进NSCLC细胞侵袭、生长、迁移过程中起重要作用。本研究旨在进一步检测HDGF在NSCLC组织标本中的表达情况，探讨其临床意义。

**方法:**

应用SP法，检测158例手术切除NSCLC及12例正常对照肺组织中HDGF蛋白表达情况，进行生存分析、预后判定。

**结果:**

与12例正常对照肺组织比较，HDGF蛋白在158例NSCLC中明显高表达（*P* < 0.001）。158例NSCLC中，HDGF高表达组（78例，占49.4%）的5年生存率为38.2%，明显低于HDGF低表达组（80例，占50.6%）的5年生存率63.1%（*P*=0.009）。直线相关分析表明HDGF表达水平与术后生存时间呈负相关（*r*=-0.183, *P*=0.022）。多因素生存分析表明术后病理分期和HDGF表达水平是手术切除NSCLC预后判定的独立因素。

**结论:**

HDGF在NSCLC中呈现高表达；HDGF高表达代表预后不良，HDGF可以作为手术切除NSCLC预后判定的新的分子标志物。

## Abstract (en)

**Background and objective:**

Our previous studies revealed that hepatoma-derived growth factor (HDGF) is highly expressed in non-small cell lung cancer (NSCLC) cells, playing important roles in promoting NSCLC cells growth and invasion. The aim of this study is to detect the expression of HDGF in 158 cases of surgically resected NSCLC and evaluate its clinical significance.

**Methods:**

Immunohistochemical SP method was used to detect the expression of HDGF in 158 NSCLC tissues and 12 normal control lung tissues. Survival analysis was further conducted.

**Results:**

HDGF was found significantly highly expressed in 158 NSCLC tissues compared with normal control lung tissues (*P* < 0.001). The 5-year survival rate was 38.2% in HDGF high expression cases, compared with 63.1% in HDGF low expression cases, the difference was statistically significant (*P*=0.009). Linear correlation analysis discovered a significantly negative correlation between HDGF expression and the survival time (*r*=-0.183, *P*=0.022). *COX* proportion hazard model analysis revealed that pathological stages and HDGF expression were independent prognostic factors for this group of 158 resected NSCLC cases.

**Conclusion:**

HDGF is highly expressed in human NSCLC tissues, predicting worse prognosis in resected NSCLCs. It might be useful molecular biomarker for predicting the prognosis of resected NSCLCs.

在世界范围内，特别是在中国，肺癌已跃居肿瘤致死的第一位，且其致死率呈逐年上升趋势^[1, 2]^。肺癌总体5年生存率仅10%-16%^[1, 3]^。在临床肺癌中，非小细胞肺癌（non-small cell lung cancer, NSCLC）约占85%，其治疗方法仍是首选以手术切除为主的综合治疗^[1, 3, 4]^。肺癌较早发生侵袭、转移是肺癌预后不佳的主要原因^[1, 3, 4]^。发现肺癌侵袭、转移的相关因子，阐明其分子机制，探讨其在肺癌诊断、预后判定乃至指导制定治疗方案中的作用，具有重要的临床意义。

近来研究表明，肝癌衍生生长因子（hepatomaderived growth factor, HDGF）可促进胎肝细胞等胚胎组织细胞^[5]^、内皮细胞、血管平滑肌细胞等增殖生长^[6, 7]^，可促进内皮细胞、血管平滑肌细胞等迁移^[6, 7]^。HDGF具有促进血管生成的作用^[6-8]^。HDGF在细胞增殖、迁移、血管生成等方面的作用，提示其可能参与肿瘤的生长、侵袭及转移过程。研究发现，HDGF在食管癌^[9]^、胃癌^[10, 11]^、肝癌^[12]^、胆管癌^[13]^、胰腺癌^[14]^以及肺癌^[15-17]^等多种人体恶性肿瘤细胞及组织中高表达，并与肿瘤侵袭转移、预后不良相关，提示HDGF在人体恶性肿瘤的发生、发展以及侵袭、转移中起重要作用，具体机制有待深入研究。

我们在前期工作^[15, 18]^中发现，HDGF在肺腺癌细胞系A549、肺鳞癌细胞系H226等NSCLC细胞系中明显高表达，应用RNA干扰技术沉默*HDGF*可以明显抑制NSCLC细胞生长、侵袭迁移能力，腹腔注射靶向*HDGF*的siRNA，可明显抑制裸鼠皮下移植瘤侵袭生长能力，应用基因芯片技术检测提示HDGF可能通过调控恶性肿瘤侵袭转移相关因子AXL^[15, 19]^、ADAM9^[15, 20, 21]^等信号转导途径发挥其分子生物学作用，揭示HDGF在促进NSCLC生长、侵袭及转移过程中起重要作用，可能是影响肺癌患者预后的重要因素。

在此基础上，本研究旨在进一步检测HDGF在中国人手术切除的NSCLC组织标本中的表达情况，验证HDGF是否在国人NSCLC组织中存在高表达，探讨HDGF表达水平在NSCLC诊断或术后预后判定中的意义，以期对指导肺癌临床工作有所裨益。

## 1 资料与方法

### 1.1 一般资料

选择2000年4月-2006年5月在中国医科大学附属第一医院胸外科接受手术治疗、具有完整病历资料及随访结果的171例NSCLC病例：术前接受B超、颅脑CT及全身骨骼核素扫描等检查排除远处器官转移，术后病理诊断为肺鳞癌或肺腺癌。排除标准：术前接受放疗和（或）化疗、术后3个月内死亡或死于非肺癌因素者。最后随访时间为2009年8月。获得有效病理切片的病例共158例，其中男性90例（57.0%），女性68例（43.0%），中位年龄60岁（31岁-79岁）。另取12例正常肺组织作为对照组。本课题研究得到中国医科大学附属第一医院伦理委员会批准；所研究病例均备有患者及家属书面签字知情同意书。

158例NSCLC手术切除方式：除少数肺周边部较小病灶病例（15例，9.5%）因年龄大或心肺功能稍差等原因行肺部分切除术（肺段切除或肺楔形切除）外，大部分病例（143例，90.5%）行标准肺癌切除术，即肺叶切除加纵隔淋巴结切除术，其中常规肺叶切除术者126例（79.7%），左或右全肺切除术12例（7.6%），袖状肺叶切除术5例（3.2%）；淋巴结切除范围：依照AJCC及Naruke提出的肺癌淋巴引流淋巴结图^[22]^，切除肺门、肺内及纵隔淋巴结^[23, 24]^。术中共切除淋巴结803组，954枚；平均5.1组/例，6.0枚/例（详见另文报告）。组织学类型：肺鳞癌45例（28.5%），肺腺癌113例（71.5%）。术后病理分期以UICC和AJCC 1997年修订的肺癌TNM分期系统为标准^[22]^：Ⅰ期82例（51.9%），Ⅱ期19例（12.0%），Ⅲ期57例（36.1%）（表 1）。术后辅助治疗：Ⅰ期患者一般不做术后辅助化疗；Ⅱ、Ⅲ期患者做4个-6个周期以铂类为基础的两药化疗；pN2患者做术后放疗（50 Gy-60 Gy）。

**1:** 手术切除的NSCLC预后因素的单因素分析结果 Univariate analysis of prognostic factors for 158 resected non-small cell lung cancer

Characteristic	*n* (%)	5-year survival rate (%)	*P*
Overall	158 (100)	51.2	
HDGF expression			0.009
Low-HDGF	80 (50.6)	63.1	
High-HDGF	78 (49.4)	38.2	
Sex			0.160
Male	90 (57.0)	48.1	
Female	68 (43.0)	55.6	
Age (year)			0.302
< 60	68 (43.0)	54.1	
≥60	90 (57.0)	49.2	
Pathological type			0.245
Squamous cell carcinoma	45 (28.5)	60.0	
Adenocarcinoma	113 (71.5)	47.8	
pTNM stage			< 0.001
Ⅰ	82 (51.9)	65.7	
Ⅱ	19 (12.0)	52.6	
Ⅲ	57 (36.1)	30.6	
T factor			< 0.001
T1	43 (27.2)	60.9	
T2	87 (55.1)	56.6	
T3-T4	28 (17.7)	20.4	
N factor			0.001
N0	92 (58.2)	60.8	
N1	21 (13.3)	50.0	
N2	45 (28.5)	32.4	

### 1.2 试剂来源

Rabbit anti-HDGF抗体^[6, 7, 15, 16]^由AD Everett教授（Johns Hopkins University）馈赠。S-P免疫组化试剂盒、DAB显色试剂盒均购自福州迈新生物技术有限公司。

### 1.3 方法

采用SP法检测HDGF蛋白在手术切除NSCLC组织标本中的表达情况。步骤如下：组织蜡块切片厚度为3 μm，常规脱蜡脱苯，梯度酒精水化，内源性过氧化物酶阻断12 min，高压抗原修复，HDGF抗体稀释比例为1:10, 000孵育过夜（4 ℃过夜），二抗体室温下孵育15 min，链霉素抗生物素蛋白-过氧化酶溶液室温下孵育15 min，DAB溶液显色1 min-3 min，苏木素复染10 min，1%盐酸酒精分化10 s，自来水浸泡20 min，返蓝，脱水，透明，封片，显微镜观察。所应用抗体的敏感性及特异性已由我们及合作者前期研究所证明^[6, 7, 15, 16]^。以PBS代替一抗作为阴性对照，每批染色切片均用同一病例已知阳性切片作阳性和阴性对照。

### 1.4 免疫组化结果判定

HDGF表达以肿瘤细胞核呈棕黄色为阳性染色。采用半定量计分方法，高倍镜下（× 400）每张切片随机选择5个视野，每个视野计数200个肿瘤细胞，计算每个视野中肿瘤细胞核染色强度和阳性染色细胞所占的百分数两者乘积，以5个视野的平均数作为每一例的核染色评分（核表达指数）。染色强度以同一张切片上阳性染色的血管内皮细胞胞核作为内对照，强度为2，若肿瘤细胞核染色强度高于血管内皮细胞核定为3，低于血管内皮细胞胞核定为1，阴性染色为0。以全部病例核表达指数的平均数作为界值，分为HDGF低表达组和高表达组。

### 1.5 数据分析和统计学处理

应用SPSS 17.0统计软件建立数据库文件并进行统计学分析。计数资料的组间比较应用*χ*^2^检验；计量资料的组间比较应用直线相关分析或t检验；单因素生存分析应用*Kaplan-Meier*法，差异的明显性应用*Log-rank*检验；多因素分析应用*COX*比例风险回归模型向前逐步回归法。以*P* < 0.05为差异有统计学意义。

## 2 结果

### 2.1 HDGF在NSCLC中的表达及其与临床病理因素的关系

HDGF在细胞浆和细胞核中均有表达。几乎所有的细胞核都有不同程度的阳性着色，包括少量淋巴细胞、成纤维细胞和血管内皮细胞。在158例NSCLC组织中，HDGF核表达平均指数为170.08±77.97；在12例正常对照肺组织中，HDGF核表达平均指数为113.33±42.07；HDGF在肺癌组织中的表达水平明显高于正常对照肺组织，差异有统计学意义（*P* < 0.001）（图 1）。

**1:**
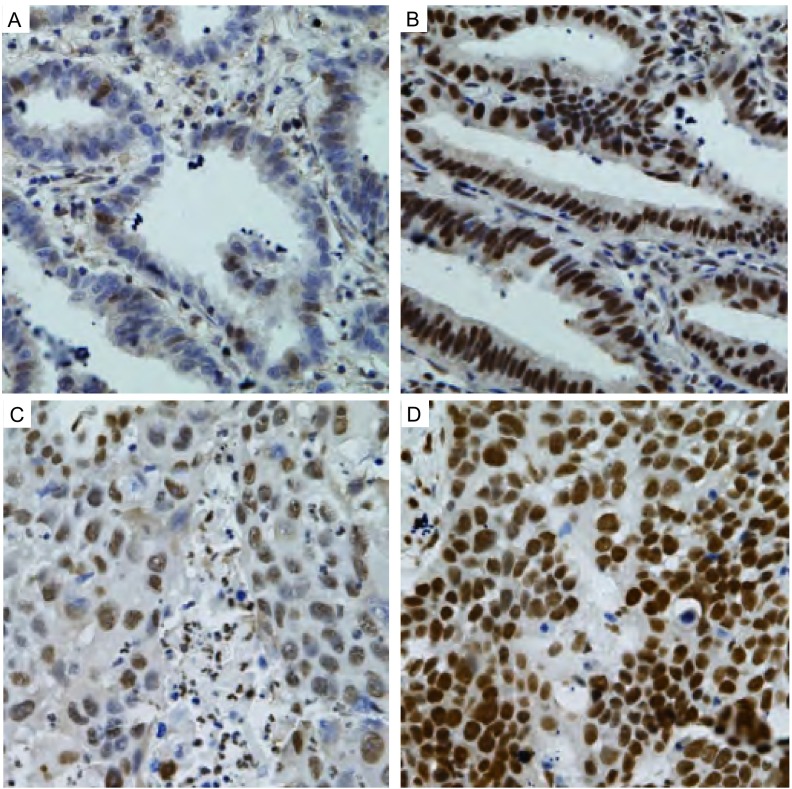
非小细胞肺癌组织中HDGF的表达（SP，×400）。A：肺腺癌中HDGF低表达；B：肺腺癌中HDGF高表达；C：肺鳞癌中HDGF低表达；D：肺鳞癌中HDGF高表达 HDGF expression in non-small cell lung cancer tissues (SP, ×400). A: HDGF low expression in lung adenocarcinoma; B: HDGF high expression in lung adeno carcinoma; C: HDGF low expression in lung squamous cell carcinoma; D: HDGF high expression in lung squamous cell carcinoma

在113例肺腺癌中，63例（55.8%）呈现HDGF高表达；在45例肺鳞癌中，15例（33.3%）为HDGF高表达；肺腺癌中HDGF高表达率明显高于鳞癌组，差异有统计学意义（*P*=0.011）。在68例女性病例中，40例（58.8%）呈现HDGF高表达；在90例男性病例中，38例（42.2%）为HDGF高表达；女性病例中HDGF高表达率明显高于男性，差异有统计学意义（*P*=0.039），而HDGF表达与年龄、病理分期、T因子及N因子等临床病理因素均无明显相关性（*P*>0.05）（表 2）。

**2:** NSCLC临床病理参数与HDGF表达的关系 Relationship between HDGF expression and clinicopathological characteristics

Characteristic	*n*	High expression of HDGF [*n* (%)]	*P*
Sex			0.039
Male	90	38 (42.2)	
Female	68	40 (58.8)	
Age (year)			0.409
< 60	68	31 (45.6)	
≥60	90	47 (52.2)	
Pathological type			0.011
Squamous cell carcinoma	45	15 (33.3)	
Adenocarcinoma	113	63 (55.8)	
pTNM stage			0.429
Ⅰ	82	38 (46.3)	
Ⅱ-Ⅲ	76	40 (52.6)	
T factor			0.322
T1	43	24 (55.8)	
T2-T4	115	54 (47.0)	
N factor			0.893
N0	92	45 (48.9)	
N1-N2	66	33 (50.0)	

### 2.2 生存分析

#### 2.2.1 HDGF表达对158例NSCLC病例预后的影响

158例手术切除的NSCLC病例，其总的5年生存率为51.2%。在158例NSCLC中，78例（49.4%）HDGF呈高表达，其5年生存率为38.2%；80例（50.6%）HDGF呈低表达，其5年生存率为63.1%；HDGF高表达组病例5年生存率明显低于HDGF低表达组，差异有统计学意义（*P*=0.009）（表 1，图 2）。

**2:**
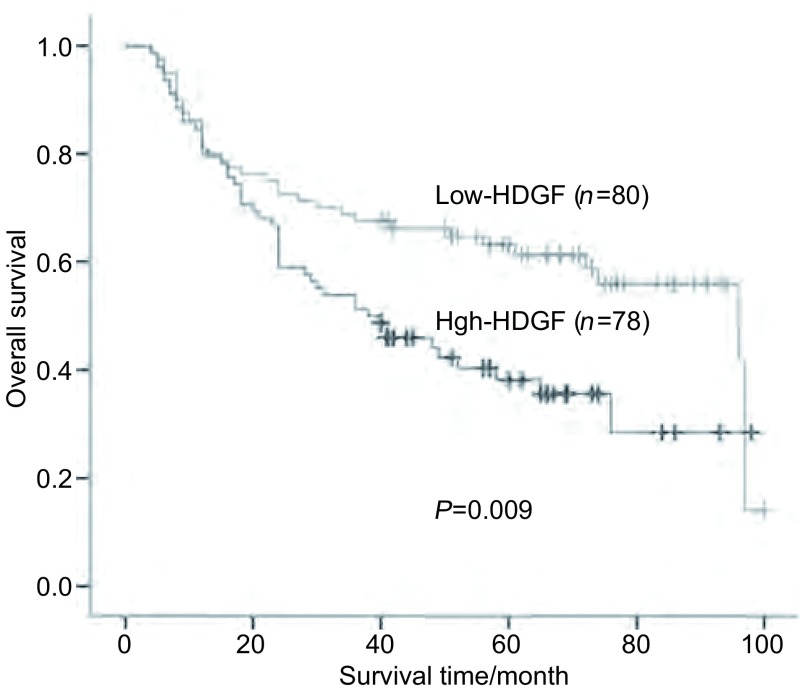
手术切除NSCLC中HDGF低表达组与高表达组的*Kaplan-Meier*生存曲线 *Kaplan-Meier* survival curves of HDGF low expression group and high expression group in 158 resected non-small cell lung cancer

应用直线相关分析方法分析HDGF表达水平与NSCLC术后生存时间的关系，结果表明手术切除的NSCLC病例术后生存时间与其癌组织中HDGF蛋白表达水平（核表达指数）之间呈负相关（*r*=-0.183, *P*=0.022）。

应用*COX*比例风险回归模型进行多因素分析，引入模型的因素有性别、年龄、手术术式、淋巴结切除个数、病理组织学类型、术后病理分期（T因子、N因子）和HDGF表达高低等，结果显示术后病理分期和HDGF表达水平可以作为手术切除NSCLC病例预后判定的独立因素（表 3）。

**3:** 对预后因素的多因素分析结果 Multivariate analysis of prognostic factors for 158 resected non-small cell lung cancer

Variate	B	SE	Wald	*P*	Exp (B)	95%CI
pTNM stage	0.530	0.119	19.799	< 0.001	1.700	1.345-2.147
HDGF expression	0.536	0.224	5.715	0.017	1.710	1.101-2.654

#### 2.2.2 HDGF在各组织学类型及病理分期中不同表达水平对生存率的影响

在45例肺鳞癌患者中，HDGF高表达组与低表达组5年生存率分别为33.3%及73.3%，差异有统计学意义（*P*=0.019）；而在113例腺癌中，HDGF高表达组与低表达组5年生存率分别为39.9%及57.1%，但差异无统计学意义（*P*=0.142）。

在82例Ⅰ期患者中，HDGF高表达组与低表达组5年生存率分别为49.6%及79.2%，差异有统计学意义（*P*=0.008）；而在76例Ⅱ期-Ⅲ期患者中，HDGF高表达组与低表达组5年生存率分别为27.7%及44.1%，但差异无统计学意义（*P*=0.342）。

在43例T1期患者中，HDGF高表达组与低表达组5年生存率分别为38.3%及84.2%，差异有统计学意义（*P*=0.011）；而在115例T2-T4期患者中，HDGF高表达组与低表达组5年生存率分别为37.2%及56.5%，但差异无统计学意义（*P*=0.088）。

在92例无淋巴结转移即N0期患者中，HDGF高表达组与低表达组5年生存率分别为44.0%及76.3%，差异有统计学意义（*P*=0.003）；而在66例有淋巴结转移（包括N1期和N2期）患者中，HDGF高表达组与低表达组5年生存率分别为30.1%及45.0%，但差异无统计学意义（*P*=0.419）。

## 3 讨论

*HDGF*基因定位于1q21-q23，有6个外显子和5个内含子，开放阅读框含720个碱基对。HDGF由240个氨基酸残基组成。HDGF的一级结构包含2个核定位序列、一个PWWP结构域。HDGF存在于细胞浆和细胞核内，主要位于细胞核内，猜测其具有转录因子的作用。HDGF的结构和功能研究正在深入进行中，HDGF可能通过其PWWP结构域与DNA等相结合发挥其特定调节功能^[25-27]^。

我们自2003年开始探索HDGF在促进NSCLC侵袭生长及转移过程中的重要作用^[15, 18]^，发现HDGF在A549等NSCLC细胞系中明显高表达，HDGF可促进NSCLC侵袭生长及迁移，应用RNA干扰技术可抑制该作用。在此基础上，Ren等^[28]^发现应用抗体策略即应用HDGF单克隆抗体可明显抑制裸鼠A549肺癌移植瘤侵袭生长能力，进一步证明HDGF在促进肺癌生长、侵袭及转移过程中的重要作用，HDGF可能成为NSCLC分子靶向治疗策略的重要的新靶标。近来Meng等^[29]^证明应用RNA干扰技术沉默*HDGF*可以明显抑制肺鳞癌细胞H520的生长、侵袭迁移能力；Zhou等^[12]^应用RNA干扰技术沉默*HDGF*可以明显抑制肝癌细胞HepG2的生长、侵袭迁移能力，均提示HDGF在促进恶性肿瘤细胞生长、侵袭转移中起重要作用，亟待深入研究。越来越多的研究证明，HDGF在食管癌^[9]^、胃癌^[10, 11]^、肝癌^[12]^、胆管癌^[13]^、胰腺癌^[14]^以及肺癌^[15-17]^等多种人体恶性肿瘤细胞及组织中高表达，并与肿瘤侵袭转移、预后不良相关。

本研究报道了中国人早期及进展期肺癌组织中HDGF高表达情况，结果显示，HDGF在手术切除的NSCLC组织中的表达明显高于正常对照肺组织，差异有统计学意义（*P* < 0.001），并且发现HDGF在肺腺癌组织中的表达明显高于肺鳞癌（*P*=0.011），与Ren等^[16]^报道美国人Ⅰ期肺癌中HDGF高表达情况一致（应用同一anti- HDGF抗体）。在女性病例中肺腺癌所占比例明显高于男性（60/69 *vs* 53/90, *P* < 0.001），而HDGF在肺腺癌组织中的高表达率明显高于肺鳞癌，因此将本研究中HDGF高表达率在性别之间的差异归因于由组织学类型构成比差异所致。

HDGF在相对早期（Ⅰ期）及进展期（Ⅱ期、Ⅲ期）NSCLC组织中均呈现高表达，其高表达率无明显差异，提示HDGF过表达出现在NSCLC发生、发展的相对早期阶段；这与Iwasaki等^[17]^报道日本人进展期肺癌组织中HDGF表达的检测结果相似；该结果尚需进一步扩大样本量并进行前瞻性研究来验证。

生存分析结果显示HDGF高表达组NSCLC病例5年生存率明显低于HDGF低表达组（38.2% *vs* 63.1%, *P*=0.009）。HDGF表达水平与肺癌患者术后生存时间呈负相关，提示HDGF高表达是影响肺癌患者生存的危险因素。

分层分析HDGF在不同病理分期中的表达情况对生存率的影响，发现在Ⅰ期、T1期和N0期NSCLC中，HDGF高表达组患者5年生存率均较低表达组明显降低（*P* < 0.05），而在进展期肺癌中HDGF高表达组的整体生存率也均有下降趋势，但差异无统计学意义（*P*>0.05），可能与影响进展期肺癌患者预后的混杂因素较多有关，尤其是受术后病理分期、主要是淋巴结转移等预后因素影响较强所致，而在受术后病理分期、主要是淋巴结转移等预后因素影响较小甚至没有影响的Ⅰ期，HDGF这一生物学因素对预后的影响作用得以彰显；与HDGF表达等生物学因素相比，术后辅助治疗的选择即术后是否放化疗以及剂量、疗程等可能也是影响进展期肺癌患者预后的更重要因素（另文专门讨论）。HDGF对进展期NSCLC预后的判定意义尚有待进一步设计更严格的对照研究来检测。

多因素分析结果显示术后病理分期和HDGF表达可以作为本组手术切除NSCLC患者预后判定的独立因素。单因素分析、多因素分析结果均表明，HDGF表达水平与术后病理分期是对手术切除NSCLC有同样明显意义的重要的预后因素，HDGF可以作为NSCLC术后预后判定的新的分子标志物。前瞻性对照研究有助于进一步验证HDGF在NSCLC术后预后判定的重要意义。

就目前而言，以解剖学特征为基础的术后pTNM分期系统，仍然是NSCLC术后预后判定的最重要依据，也是决定术后辅助治疗如术后是否放化疗的最重要依据^[22, 30]^，但在临床实践中仍有很大的局限性、面临很大的困惑，即相同术后pTNM分期可能有着完全不同的预后，如活过5年（至观察时限，本研究中仍存活者中最长生存时间为100个月）或不能活过5年（本组最短生存时间为4个月），即使是选择相同术后治疗方案、而且性别、年龄、病理类型、手术术式等等完全配对相同的患者也可能同样有着完全不同的预后，若能把一些重要的生物学因素如HDGF表达高低等等考虑进来，就可能更好地解释这种区别的由来，即由于肿瘤个体最本质的生物学特性不同所致。

本研究中，158例手术切除NSCLC病例，其总的5年生存率为51.2%；与超过一半的肺癌手术患者（51.2%）活过5年以上相对应，有约近一半肺癌手术患者（48.8%）死于术后5年内，其中大量存在这样的情况，即相同术后pTNM分期、选择相同术后治疗方案，但有着不同的预后。这种区别不能用术后pTNM分期、选择相同术后治疗方案来解释，更不能用性别、年龄、病理类型、手术术式、淋巴结切除个数等对术后预后影响更弱的因素来解释（依据多因素分析结果）。

若考虑到HDGF表达高低这一生物学因素，在158例NSCLC中，HDGF低表达组5年生存率为63.1%，而HDGF高表达组5年生存率明显降低为38.2%，差异有统计学意义（*P*=0.009），就总体而言，HDGF表达高低这一生物学因素可能是与术后pTNM分期强度近似的预后指标，即如果某一手术切除的肺癌病例HDGF低表达，有63.1%机会活过5年，几乎与本研究中术后Ⅰ期肺癌患者的5年生存率相同，也与文献^[22, 31]^中所报道的术后Ⅰ期肺癌患者的5年生存率相当；而一旦HDGF高表达，则仅有38.2%的机会活过5年，更接近于本研究中术后Ⅲ期病例的5年生存率，与文献^[22, 31]^中所报道的术后Ⅲa期或Ⅱb期肺癌患者的5年生存率相当；结合多因素分析结果，足以说明HDGF表达高低这一生物学因素是除了术后pTNM分期外比其它通常指标更好的、更强的预后指标。没有理由不考虑把HDGF表达这样的对肺癌患者预后有明显影响的生物学因素加入现有的TNM分期系统。

分层分析提示，在82例Ⅰ期患者中，HDGF高表达组与低表达组5年生存率分别为49.6%及79.2%，差异有统计学意义（*P*=0.008），表明如果某一Ⅰ期肺癌病例HDGF低表达，则有近80%机会生存期超过5年，而一旦HDGF高表达，则生存期少于5年的机会超过50%。

因此，若能在原有TNM分期基础上加上生物学因素分期如Ⅰ期-HDGF(+)组及Ⅰ期-HDGF(-)组，无疑将增加术后分期的准确性，增加据此分期进行术后预后判定的准确性，更重要的是，这一生物学因素指标可能成为指导术后辅助治疗选择的更有效的预测指标，如本研究Ⅰ期病例中，HDGF高表达组5年生存率明显降低为49.6%，更接近于通常术后pTNM分期Ⅱ期患者的5年生存率^[22, 31]^，显而易见，无论该患者更细分为Ⅰa还是Ⅰb期，都应该进行术后辅助治疗如化疗等，而对于HDGF低表达组，其5年生存率接近80%，如果没有更好的其它生物学预后指标进行再分期以找到进一步的预后差别，那么这一组HDGF低表达病例就没有必要进行术后辅助治疗如化疗，以避免不必要的化疗毒性。已有研究^[32]^证明，术后化疗的毒性作用对Ib期肺癌病例可能是长期的、致命的。因此，如果能发现更多的像HDGF这样的生物学预后指标联合应用，加入术后pTNM分期系统，即加入患者个体生物学因素亚分期，将更好地完善术后pTNM分期系统，更准确地进行预后判定，并更大价值地用于指导术后辅助治疗如化疗方案、剂量、疗程等的选择，即施行真正的基于患者个体、肿瘤组织本身分子生物学本质特性的个体化治疗方案，并更准确地进行疗效预测。这也正是近年来不断呼吁将肺癌患者个体生物学因素加入术后pTNM分期系统的真正意义所在。

最近，Zhu等^[33, 34]^报道检测某些特定生物学标志物mRNA表达水平，从而将肺癌病例分为高风险组和低风险组，高风险组病例接受术后化疗可能受益，而低风险组患者接受术后化疗无益处，亦提示在现有术后pTNM分期系统基础上加入生物学因素亚分期，具有重要的实际临床指导意义。有待于发现更多的、更有效地分子生物学指标，广泛验证，阐明其分子机制，以指导临床医生制定更合理、有效的肺癌术后辅助治疗方案，提高治疗肺癌的能力。

到目前为止，HDGF的分子机制尚不很清楚。深入研究、阐明HDGF促进NSCLC细胞侵袭生长、转移的分子机制，发现更多的、更有效的分子标志物，从中筛选并组合应用，将为应用HDGF等生物学指标作为手术切除NSCLC术后预后不良的分子标志物、并进一步作为预测标志物指导术后治疗策略的选择等提供更坚实的理论依据；也将为靶向HDGF的NSCLC分子靶向治疗的开展提供更广阔的前景、更明确的路径。
